# Examining the Deviation From Balanced Time Perspective in the Dark Triad Throughout Adulthood

**DOI:** 10.3389/fpsyg.2018.01046

**Published:** 2018-06-26

**Authors:** Béla Birkás, András Matuz, Árpád Csathó

**Affiliations:** Institute of Behavioral Sciences, Medical School, University of Pécs, Pécs, Hungary

**Keywords:** balanced time perspective, Dark Triad, adulthood, life history, adaptation

## Abstract

Individuals who score high on Dark Triad (DT) personality traits (i.e., Machiavellianism, subclinical narcissism and subclinical psychopathy) have been found to prefer a fast life strategy with enhanced motivation for immediate resource acquisition and short-term benefits. In line with these points, recent studies have found evidence showing that DT traits are associated with a biased, strongly present-oriented time perspective. In the current study, we aimed to examine whether the temporal attitude of individuals high in DT is deviant from a balanced time perspective (BTP) to a significant extent. To achieve this aim, we applied two operationalizations published in earlier studies to quantify BTP: the Deviation from Balanced Time Perspective coefficient (DBTP), calculated as the difference between individuals’ time perception and the optimal time perspective, as well as the person-oriented approach of identifying groups of individuals with similar time perception. Importantly, the age of participants (*N* = 346) covered a long and continuous period of adulthood—from the young adulthood to the elderly—in order to examine the moderating effect of age on the association of DT and BTP. Machiavellianism and psychopathy were both found to be clearly deviant from a BTP. In contrast, higher scores on narcissism were positively associated with a BTP profile. The DBTP analysis, however, suggested that this beneficial effect of narcissism was only prevalent among the elderly individuals.

## Introduction

Very soon after birth, time becomes one of the most important decision markers used to make either implicit or explicit strategic decisions about how we organize and prioritize future goals (Boniwell and Zimbardo, 2015). One well-known psychological concept dealing with variability in views about the past, present and future is that of time perspectives (TP). An extensively investigated model of TP was developed by Zimbardo and Boyd (1999) and operationalized in the Zimbardo Time Perspective Inventory (ZTPI). The model conceptualizes the psychological dimension of time as a result of cognitive operations by which personal and social experiences over time are automatically enrolled into time frames of the past, present and future (Zimbardo and Boyd, 1999). In line with this, the ZTPI defines TP in terms of five factors, where each factor reflects a different view of time: past-negative, past-positive, present-fatalistic, present-hedonistic, and future-oriented. Thus, the attitudes toward both the past and the present frames of time are assessed by two subscales addressing positive and negative views about the past and present separately. In contrast, items related to future frame were found to be related to a single factor that reflects a general future orientation attitude in strong association with striving for future goals and reward (Zimbardo and Boyd, 1999).

It has further been proposed that in order to achieve an optimal, adaptive, functioning life, these different TP should be integrated into a balanced profile (Zimbardo and Boyd, 2008). In other words, a balanced time perspective (BTP) is suggested as being an optimal alternative to being restricted to one particular time perspective (Zimbardo and Boyd, 2008; Boniwell and Zimbardo, 2015). BTP is usually operationalized in terms of the ZPTI as moderate-to-high scores on the past-positive, present-hedonistic and future perspectives, and low scores on the past-negative and present-fatalistic perspectives. The concept of BTP has received support from many studies showing that when the attitude toward time cannot be characterized by an excessive orientation toward a specific perspective, in particular for the negative orientations (e.g., past-negativity, and present fatalism) then that might have a beneficial effect on well-being, mental health, and psychosocial functioning. For example, a study by Drake et al. (2008) showed evidence that, of their participants, the happier and more mindful individuals were those who had a BTP profile. Similarly, Boniwell et al. (2010) discovered four different time perspective clusters (future-oriented, present-oriented, balanced, and negative TPs) in student samples, and found that the highest well-being scores were represented in the balanced TP cluster. The findings of other studies called attention to the positive association between imbalanced TP and risk factors for the development of pathological substance use. Thus, investigating a large sample of adolescents, engagement in binge eating and drinking was more frequent among those who were concerned less about the future, had a more negative attitude toward the past, and had higher level of present fatalism (Laghi et al., 2012). Keough et al. (1999) also found evidence that individuals having higher present orientation reported frequent substance use (i.e., alcohol, tobacco, and other drug use).

Besides the advantages of a BTP profile, however, it has also been suggested that changes in the environment might require a shift from the ideal time perspective balance (Zimbardo and Boyd, 2008). For example, where environmental circumstances are predictable, and social and material resources constantly available, the situation is ideal for future-oriented behavior by which the duration of this environmentally stable period can be maximized. In contrast, when environmental conditions become uncertain, a more pronounced present-oriented time perspective might be a more adaptive choice because robust orienting to the present can potentially provide the greatest capacity for immediate resource acquisition and storage.

Present-oriented TP is probably one of many behavioral attitudes and traits that underpin a fast life strategy. According to Life History Theory (LHT), fast life strategists are those who, possibly as a consequence of an unpredictable environment experienced during their childhood, favor behaviors that require minimal investment but offer fast gratification (Buss, 2015). Evidence shows that they tend to prefer risk-taking behavior (Griskevicius et al., 2011), experience an earlier start to and higher frequency of mating, and invest relatively little in social relationships (Belsky et al., 1991). Furthermore, certain personality traits appear to be reinforced by unpredictable environments (Jonason et al., 2017) and thus are associated predominantly with a fast life strategy. Specifically, individuals with Dark Triad personalities (DT) have been found to strongly prefer behaviors associated with a faster life strategy (Jonason et al., 2010). The DT includes three interrelated constructs, namely, Machiavellianism, subclinical psychopathy and subclinical narcissism (Paulhus and Williams, 2002). The fast life strategy preference is associated by many common features of the three traits: callousness, manipulation (Jones and Paulhus, 2011), a low level of honesty-humility (Lee and Ashton, 2005), diminished self-control (Jonason and Tost, 2010), selfishness, inability to delay gratification (Brumbach et al., 2009; Birkás et al., 2015) and exploitation (McDonald et al., 2012). A logical assumption to take from the above points is that the predominantly fast life strategy of individuals high in DT traits goes along with a negative-past perspective and a strong present orientation. This assumption was clearly supported in our earlier study, which showed that all three DT traits were related to present-oriented perspectives and, in addition, that Machiavellianism and psychopathy were associated with a negative, dispirited view of the past (Birkás and Csathó, 2015). Since then, three other studies have drawn similar conclusions. First, Stolarski et al. (2017) investigated associations between the DT and TP, predicting that sociosexuality as a marker of life history strategy (i.e., higher sociosexuality indicates faster life strategy) would mediate associations between these two domains. Their findings suggested that individuals with a stronger psychopathic or Machiavellian trait tend to have a predominantly negative view of the past and a stronger focus on the present. A partial mediation effect of sociosexuality on the TP–DT relationship in females was also found. Second, Moraga et al. (2017) further investigated the differential effect of gender on the association of the DT with TP. On one hand, they replicated the finding that present-focused and past-negative biased TP is more frequent in individuals with higher DT traits; on the other, they found no evidence of a moderating effect of gender on this association. Third, Jonason et al. (2018) study provided insights into country-wide variations in TP–DT associations. Based on samples collected from three countries (Australia, Japan, and Russia) they found that, unlike narcissism, psychopathy and Machiavellianism were associated with a negative view of the past and limited concern about the future. Interestingly, a moderation effect of country was also demonstrated, mainly in terms of temporal preference of individuals higher on narcissism: narcissism was found to be associated with less future concerns in Russia and Australia, and more future concerns in Japan (Jonason et al., 2018).

In the present study, we aimed to further examine the nature of the association between TP and the DT traits. The study was inspired by two points in particular. First, in our previous study, although covariate-controlled regression analyses suggested a reliable bias in TP for high-scoring DT individuals, this finding was only indirectly indicated by the results because there was no direct operationalization of individual differences in temporal perspective bias. Previous studies have suggested the use of different operationalizations to quantify the magnitude of imbalance in individuals’ temporal preference. See, for example, the methods reported in Boniwell et al. (2010) and Zhang et al. (2013b). The three studies described in the preceding paragraph did not apply these operationalizations either. These methods were, however, given considerable weight in the analyses of the current study.

The second reason behind this study was the interest in the potential moderating role of age in DT-BTP associations. The findings of many previous studies have highlighted that the age of individuals has a significant impact on their TP. Looking only at the most robust effects, older adults tend to evaluate their past with more satisfaction than do people of a younger generation; compared with the young, elderly individuals also attribute lower importance to a pleasure-seeking, hedonistic outlook on life (for a recent review see, e.g., Laureiro-Martinez et al., 2017). These changes in TP across life-stages are thought to be underpinned by a change in life focus: as people get older they prioritize materialistic goals less and emotion-related goals more—that is, they tend to live in accordance with a slower life strategy. One might assume that such changes in TP during the course of adulthood might also have an influence on the associations between TP and the DT; as individuals get older, even those with high scores on the DT traits might look back with an emotionally more positive attitude. Such attitudinal change in the elderly might in turn result in a smaller deviation from the optimal level of the past-negative dimension of TP for older individuals high on the DT traits. Similarly, given the generally lower level of hedonism in older age, as individuals with pronounced DT traits age, they might move closer to the ideal level.

In order to examine the potential moderating role of age in DT-BTP associations, we collected data on a wide age-range of individuals, spanning from young to late adulthood. The dataset was also varied in terms of participants’ education, further improving the generalisability of the current study’s findings.

## Materials and Methods

### Participants

Participants encompassed 346 adults (100 men, 246 women) aged between 18 and 85 years (*M* = 43.34, *SD* = 20.46). The majority of the participants were educated to high-school level or above (88%). As a limitation of the study, higher education level was biased in terms of age: almost all the younger participants were either college undergraduate or graduate students, or had an advanced education degree (education was assessed on a six-point scale). Therefore, all analyses reported in this study controlled for education level. The majority of the participants were either students or employed, while 102 participants were retired. Data from the younger individuals (<30 year) were collected entirely by an online measurement. The data from the other participants were collected as part of a larger study examining the mental and physical health of middle-aged and elderly individuals. The data collection started with an online measurement but this reached only a few elderly individuals. Therefore, printed questionnaires were distributed with the help of local Pensioners Clubs. Participants were asked not to complete the questionnaires while in the Clubs or within any organized team program.

### Measures and Procedure

To assess the DT construct the Short DT questionnaire was used (SD3; Jones and Paulhus, 2014). The SD3 is a 27-item self-report scale designed to measure the subscales of Machiavellianism, Psychopathy and Narcissism. Each subscale consists of nine items presented on a 5-point scale. In the current sample, the removal of item 21 from the narcissism subscale meaningfully improved internal consistency (the item-total correlation was strongly negative); therefore this item was excluded from the scoring of narcissism. The mean score of each subscale was calculated and used in the analyses. The descriptive statistics for the SD3 are shown in **Table 1**. Internal consistencies (i.e., Cronbach’s α) were acceptable: Machiavellianism = 0.66; Psychopathy = 0.68; Narcissism = 0.73. In the first validation studies of SD3, Jones and Paulhus (2014) reported internal consistencies ranged between 0.71 and 0.76 for Machiavellianism, 0.72 and 0.77 for psychopathy, and 0.68 and 0.78 for narcissism.

**Table 1:** Descriptive statistics of the Demographic, Dark Triad, and Time perspective variables.

Variables	Mean	*SD*
Age	43.34	20.46
Education	4.31	1.18
*Dark Triad traits*		
– Machiavellianism	3.09	0.63
– Narcissism	2.70	0.75
– Psychopathy	2.03	0.61
*Time perspectives*		
– Past-negative	2.67	1.10
– Past-positive	3.42	1.01
– Present-hedonism	2.59	1.02
– Present-fatalism	2.84	1.08
– Future	3.95	0.88
*DBTP*	3.13	0.90
*DOTP*		
– Past-negative	1.04	0.80
– Past-positive	1.26	0.90
– Present-hedonism	1.44	0.82
– Present-fatalism	1.41	0.98
– Future	0.71	0.52

*N = 346; men: 29%, women: 71%; Age range continuous: 18 – 85 year, median = 38; DBTP, Deviation from the Balanced Time Perspective calculated based on Stolarski et al. (2011). DOTP, the absolute deviation from the optimal value for each scale separately.*

In addition, to assess participants’ TP, we used the short form of the Zimbardo Time Perspective Inventory (ZTPI-short, 17 items). The ZTPI-short was used because of concerns about overall completion time. Except for the younger adults, the participants were taking part in a larger project comprising many other questionnaires, and therefore the intention was to keep the overall duration of completion relatively short in order to minimize participants’ fatigue and loss of motivation. In their questionnaire battery, the ZTPI-short came toward the beginning of the battery, immediately after some demographic questions and immediately before the SD3. Accordingly, responses were unlikely to have been influenced greatly, if at all, by any other questionnaires in the battery. A description of the ZTPI-short follows.

The validated Hungarian version of the ZTPI-short is a 17-item self-report measure, with five subscales covering cognitive, motivational and emotional aspects of past, present and future TP on a five-point scale (Zhang et al., 2013a; Orosz et al., 2017). The following five factors are differentiated in ZTPI-short: the Past-negative factor, characterized by a focus on past failures and frustrations (e.g., ‘I’ve taken my share of abuse and rejection in the past’); the Past-positive factor, reflecting positive attitudes toward past events (e.g., ‘Happy memories of good times spring readily to mind’); the Present-hedonism factor, referring to a hedonistic, pleasure-seeking attitude that ignores future consequences; the Present-fatalistic factor, defined by the belief that events are predetermined by external forces and so the future is pre-ordained, such that individuals with a high present-fatalistic attitude are resigned to present events (e.g., ‘My life path is controlled by forces I cannot influence’); and, the Future factor, on which high-scoring individuals focus on planning for future goals and accept delays in gratification in order to achieve better outcomes from long-term actions (e.g., ‘I’m able to resist temptations when I know that there is work to be done’). Mean scores were calculated for each time perspective subscale, and were used in the analyses. Descriptive statistics for ZTPI-short are also shown in **Table 1**. Internal consistencies (i.e., Cronbach’s α) were adequate: Past-positive = 0.72; Past-negative = 0.81; Present-hedonism = 0.72; Present-fatalism = 0.69; Future = 0.78.

### Analysis

To analyze the associations between the dimensions of the DT and the deviation from a BTPs, three main analyses were performed.

[a] First, we calculated deviation from an ideal, DBTP based on the model suggested by Stolarski et al. (2011):

$$\begin{array}{ccc} DBTP & = & \sqrt{{\left(oPN-ePN\right)}^{2}+{\left(oPP-ePP\right)}^{2}+{\left(oPF-ePF\right)}^{2}+{\left(oPH-ePH\right)}^{2}+{\left(oF-eF\right)}^{2}} \end{array}$$

Specifically, Stolarski et al. (2011) suggested that there is an optimal point on each time perspective, and that deviation from this optimal point in any direction impairs individuals’ experience of subjective functionality and well-being. The optimal values they defined for each scale were based on Zimbardo and Boyd’s proposal^1^ and cross-cultural database, and were as follows: for the Past-positive scale, a score of 4.60 (oPP); for the Past-negative scale, a score of 1.95 (oPN); for the Present-hedonism scale, a score of 3.90 (oPH); for the Present-fatalism scale, a score of 1.50 (oPF); and for the Future scale, a score of 4.00 (oF) (see Stolarski et al., 2011 for a more detailed explanation). To calculate the DBTP for each time perspective scale, the empirically determined scores were substracted from the optimal scores and the resulting differential scores combined, as shown in the equation above. The DBTP values close to zero indicate a more BTP. In this analysis, two multivariate regression models were tested. In the first model, the dependent variable was the DBTP scores, and the three DT scales were entered as predictors together with the demographic variables (age, gender, education). The second regression model consisted of a second predictor block of three interaction terms to assess whether participants’ age moderated the TP-DT associations. More specifically, three two-factor interaction terms (one for each DT scale, as for example the age × Machiavellianism interaction) were calculated using *Z* scores. In the present study, we aimed at revealing gender-independent two-way interactions, therefore, the significant two-way interactions were probed for gender-specificity. Specifically, if a two-factor interaction was found to be significant, then we tested whether this interaction was gender-independent by using a three-factor interaction term (e.g., age × Machiavellianism × gender). To probe the gender specific effects, the second model of the analysis was performed again by adding the three-factor interaction term to the block of predictors. Gender specificity was taken into account, because it is a well-established finding in former studies that men usually score higher on the DT scales than women (see e.g., Kruger et al., 2008; Jonason et al., 2010). In addition, there is also evidence for the different time-related attitudes of the two genders (Mello and Worrell, 2006). Consequently, the age related changes in the association of the DT and the TP might occur differently in the two genders. Each significant interaction was analyzed further by simple slope analysis (Aiken et al., 1991).

[b] Second, to probe the unique contribution of the DT sub-scales predicting the deviations of the different TP we calculated the absolute deviation from the optimal value for each scale separately (henceforth DOTP-subscale, e.g., DOTP*_past-negative_*: |o*PN* – e*PN*|; DOTP*_past-positive_*: |oPP – ePP|. Again, two regression models were tested. In the first model, one block of predictors with the three DT variables as well as the demographic variables. In the second, similarly to the first analysis, the two-factor interaction terms were used as predictors in a second block of the model. The moderating role of gender in the significant two-way interactions was again probed in an additional analysis as elaborated above.

[c] Third, to determine the group of individuals who have a BTP we adopted the person-oriented approach suggested by Boniwell et al. (2010). The person-oriented approach can be assessed not only to identify the group of individuals with BTP, but it can also be applied to identify individuals with a specific TP profile. More specifically, a hierarchical cluster analysis was carried out using standardized scores, Ward’s method and the Squared Euclidean metric to identify groups of participants with similar TP patterns in the sample. We performed the analysis with two to five clusters, and selected the cluster result based on two criteria: first, the TP profile for the cluster results had to be in line with the profiles suggested by Zimbardo and Boyd (2008); and second, the between-cluster difference had to reach significance for the DT traits (using a one-way ANOVA). We selected the five-cluster result of the analysis because that showed the best fit with these criteria.

In addition to these three main analyses, we performed a separate series of multiple regressions to explore the association of age with DT scales, controlling for education and gender.

## Results

**Table 1** presents descriptive statistics for the demographic variables, the DT and the time perspective scales. Analysis of the association of age with DT dimensions revealed a significantly negative association of age with all three DT dimensions: with increasing age, participants reported a lower level of Machiavellianism (*β* = -0.19, *t* = -2.82, *p* < 0.001), narcissism (*β* = -0.49, *t* = -7.39, *p* < 0.001) and psychopathy (*β* = -0.46, *t* = -7.4, *p* < 0.001). In addition, participants’ gender and education level were significantly associated with the reported levels of Machiavellianism and psychopathy. Specifically, scores of Machiavellianism and psychopathy decreased as a function of education level (*β* = -0.46, *t* = -2.04, *p* < 0.05), and men reported significantly higher level of these two DT traits than women did [Machiavellianism: *t*(344) = 3.83, *p* < 0.001; psychopathy: *t*(344) = 3.56, *p* < 0.001]. For narcissism, no similar associations with gender and education were found.

**Table 2** shows the results of the first main analysis. The analysis revealed that deviation from an optimal balanced time perspective profile (i.e., DBTP) is significantly predicted by each of the three Dark Triad traits.

**Table 2:** The results of the multivariate regression analyses: associations between the Deviation from Balanced Time Perspective (DBTP) and the Dark Triad; *standardized* β *and R^2^ values*.

Predictors	β	
	DBTP	
	M. I.	M. II.
**First block**		
Age	0.20**	0.19**
Gender	0.13*	0.12*
Education	0.04	0.05
Machiavellianism	0.16**	0.14*
Narcissism	-0.26**	-0.28**
Psychopathy	0.15*	0.15*
**Second block (interaction terms)**		
Age × Machiavellianism	–	0.08
Age × Narcissism	–	-0.17**
Age × Psychopathy	–	0.06
R^2^	0.13**	0.16**

*^∗^p < 0.05; ^∗∗^p < 0.01; M. I., Modell I.; M. II., Modell II.*

These results are in line with expectations for Machiavellianism and psychopathy, because participants with high scores on these two scales also exhibited greater deviation from BTP. The analysis also revealed a significant DBTP – narcissism association but the trend of this association was negative. This finding suggests that participants with a stronger narcissistic personality deviated less from a BTP profile than did participants with low scores on narcissism. This association was, however, found to be moderated by individuals’ age. For the source of this interaction, the slope analysis indicated that there was a significant negative association between narcissism and DBTP for older adults (i.e., at 1 SD above the mean age; *β* = -0.31, *t* = -3.07, *p* < 0.01), but not for younger adults (i.e., at 1 SD below the mean age; *β* = -0.09, *t* = -1.03, *n.s.*). As the second analysis below indicates, this negative association was underpinned by lower deviation from the optimal value in present-hedonism, and present-fatalism for older individuals having a higher narcissistic character. In addition to the DT traits, participants’ gender was also found to predict DBTP: women had higher deviation from BTP than men had. As the analysis of the three-factor interaction of age × narcissism × gender revealed, predictability of the interaction of age × narcissism on DBTP was not different for the two genders (*β* = 0.09, *t* = 0.85, *n.s.*).

The results of the second main analysis are shown in **Table 3**. In the first models, analysis of the DT traits as predictors revealed that higher Machiavellianism predicts a larger deviation from optimal present-fatalism. In the second models of the analysis, the interaction between age and Machiavellianism predicted the deviation from future time perspective only. As the results of the slope analysis suggested this interaction, however, was non-interpretable: it was underpinned by opposite but non-significant trends both for the younger (i.e., at 1 SD below the mean age; *β* = -0.07, *t* = -0.88, *n.s.*) and the older (i.e., at 1 SD above the mean age; *β* = 0.19, *t* = 1.58, *n.s.*) adults. The effect of gender on this interaction was also non-significant (*β* = 0.03, *t* = 0.34, *n.s.*).

**Table 3:** The results of the multivariate regression analyses: associations between the Dark Triad and the absolute deviation from an optimal level of time perspective in each time perspective subscale (DOTP); *standardized* β *and R^2^ values*.

Predictors	β									

	DOTP-subscales									

	Past-negative		Past-positive		Present fatalism		Present hedonism		Future	

	M. I.	M. II.	M. I.	M. II.	M. I.	M. II.	M. I.	M. II.	M. I.	M. II.
**First block**									
Age	0.01	0.02	-0.09	-0.06	0.36**	0.35**	0.06	0.02	-0.01	-0.05
Gender	0.01	0.10	-0.04	-0.04	0.06	0.06	0.13*	0.11*	-0.04	-0.03
Education	-0.05	-0.05	0.24**	0.26**	-0.05	-0.03	-0.03	-0.04	0.00	0.00
Machiavellianism	0.07	0.08	-0.07	-0.06	0.19**	0.18**	0.06	0.02	0.01	0.01
Narcissism	-0.11	-0.1	0.02	0.04	-0.21**	-0.22**	-0.25**	-0.30**	-0.03	-0.05
Psychopathy	0.25**	0.25**	0.02	0.04	0.13*	0.14*	-0.15*	-0.15*	0.15*	0.14m
**Second block (interaction terms)**										
Age × Machiavellianism	–	0.03	–	0.01	–	0.10	–	-0.05	–	0.12
Age × Narcissism	–	0.05	–	0.01	–	-0.15**	–	-0.22**	–	-0.02
Age × Psychopathy	–	0.07	–	0.15*	–	0.05	–	0.08	–	-0.20**
*R*^2^	0.06**	0.07**	0.11**	0.13**	0.26**	0.29**	0.17**	0.21**	0.03	0.06*

*^∗^p < 0.05; ^∗∗^p < 0.01; m: p = 0.05; M. I., Modell I.; M. II., Modell II.; Model I: one block of predictors was entered; Model II. included a second predictor block of three interaction terms.*

Deviation from the optimal TP, however, was even more pronounced even more pronounced for psychopathy: psychopathy predicted higher values on three of the five DOTP-subscales. Specifically, deviation from the optimal values for past-negativity, present fatalism, and future time perspective were positively associated with scores for psychopathy. In contrast, psychopathy significantly but negatively predicted the deviation from the optimal level of present hedonism. This negative association can be explained by a special distribution of present-hedonism scores in the dataset. Most of the individuals (∼76%) in this sample had a suboptimal score on present hedonism. Thus, the negative association between DOTP*_present-hedonism_* and psychopathy suggests that the increased level of hedonism characterizing individuals with high scores on psychopathy might still be beneficial because that level is close to the optimal one suggested for this TP.

Of the interactions with age, DOTP*_past-positive_* and DOTP*_future_* were found to be predicted by the age × psychopathy interaction. The association of this interaction with DOTP*_past-positive_* was again non-interpretable: as the source of this significant interaction, the trends for younger (i.e., at 1 SD below the mean age; *β* = -0.12, *t* = -1.26, *n.s.*) and older (i.e., at 1 SD above the mean age; *β* = 0.17, *t* = 1.35, *n.s.*) adults had opposite directions, but both of these trends were found to be non-significant by separate analyses. In contrast, the slope analysis of the significant association between DOTP*_future_* and the age x psychopathy interaction revealed that the association of DOTP*_future_* and psychopathy predicts a non-optimal level of future orientation in younger adults only (i.e., at 1 SD below the mean age; *β* = 0.29, *t* = -3.25, *p* < 0.05). No age-related changes in future orientation were found, but scores of psychopathy showed a quite strong decline with increasing age. Such a low level of psychopathy might no longer be associated with a disadvantageous level of future orientation. None of the significant two-factor interactions was found to be gender-specific (DOTP*_past-positive_* - age × psychopathy × gender: *β* = 0.06, *t* = 0.60, *n.s.*; DOTP*_future_* - age × gender × psychopathy: *β* = 0.04, *t* = 0.45, *n.s*).

Similarly to psychopathy, narcissism significantly and negatively predicted the deviation from the optimal present-hedonism level. In addition, the association of DOTP*_present-fatalism_* and narcissism was significant: participants scoring high on narcissism were found to report a more optimal level of present-fatalism compared with participants with a less narcissistic personality. The results obtained in the second model of the analysis, however, implied that these associations were moderated by participants’ age. Exploring the source of these interactions, it was found that the association between DOTP*_present-hedonism_* and narcissism was significant in the older adults only (i.e., at 1 SD above the mean age; *β* = 0.29, *t* = -3.25, *p* < 0.05). Furthermore, it was found that the DOTP*_present-fatalism_* - narcissism association has a steeper slope in the older (i.e., at 1 SD above the mean age; *β* = -0.39, *t* = -3.58, *p* < 0.01) than in the younger (i.e., at 1 SD below the mean age; *β* = -0.19, *t* = -02.32, *p* < 0.05) adults. The descriptive statistics for present hedonism (*M* = 2.29, *SD* = 0.99) and present fatalism (*M* = 3.48, *SD* = 1.05) in older adults suggest that elderly individuals had a higher than optimal fatalistic attitude, and a lower than optimal hedonistic attitude. In addition, as is shown in **Table 3**, deviation from the optimal level of present fatalism increased as a function of participants’ age. Consequently, the negative association of narcissism with DOTP*_present-fatalism_* and DOTP*_present-hedonism_* in older adults suggests that narcissistic personality might have a compensatory effect on the suboptimal level of these TPs in elderly people. Again, no effect of participants’ gender was identified on the significant two-way interactions (DOTP*_present-fatalism_* - age × gender × narcissism: *β* = -0.06, *t* = -0.71, *n.s.*; DOTP*_present-hedonism_* - age × gender × narcissism: *β* = 0.05, *t* = 0.47, *n.s.*).

Of the demographic variables, gender was found to have a significant effect on the deviation from the optimal level of present hedonism only: Women tended to report a less optimal level of hedonism than men. Education also had one effect only: It was associated positively with DOTP*_past-positive_*, suggesting that participants that are more educated reported a less positive attitude about the past. In line with this, a correlation analysis showed that participants reporting a higher level of education had significantly lower past-positive scores (*r* = -0.35, *p* < 0.001). Because education level was biased in terms of age, we calculated an age × education interaction, and performed the regression analysis again including this interaction term. No significant association of age × education with DOTP*_past-positive_* was obtained, implying that the association between DOTP*_past-positive_* and education is independent from participants’ age (*β* = -0.01, *t* = -0.1, *n.s.*).

Cluster means and standard deviations of the hierarchical cluster analysis (i.e., the third main analysis) are shown in **Table 4**. The five clusters can be characterized by the following person-based temporal profiles. The profile of Cluster 5 can be interpreted as a cluster with BTP profile, where future-orientation is the most dominant perspective, but the past-positive and present-hedonistic perspectives are also represented with higher values than past-negativity and present-fatalism. These scores on past-negativity and present-fatalism have the lowest mean in Cluster 5. All the other clusters appear to have an imbalanced temporal profile. In Cluster 1, the mean score of past-negativity is low along with high scores in the past-positivity and future orientation. The temporal profile, however, remains imbalanced because present-hedonism has the lowest mean score of all the clusters, and present-fatalism achieved a very high score. Similarly, in Cluster 2, three TP appear to be in line with an optimal profile. Thus, future-orientation and past-positivity are represented with high values – these TPs have higher means than their means across the clusters (i.e., grand mean). Still in line with an optimal TP profile, present fatalism was found to be low, with a lower mean than its grand mean. However, the salient characteristics in this cluster also are the high mean score of past-negativity (i.e., it is much higher than its grand mean), and the relatively low score of present hedonism, making this cluster different from an optimal profile. Cluster 3 is without a dominant time perspective character: The five time-perspectives have similar mean values, suggesting that individuals belonging to this cluster have also a different-to-optimal TP profile. Specifically, the past-negative TP reaches the highest, and the future-orientation TP reaches the lowest mean in comparison with other clusters. The mean score of present fatalism is also far above its grand mean, strengthening the non-optimal profile of this cluster. In contrast, the relatively high level of past-positive and present-hedonism TPs seem to partially optimize the profile of this cluster. Finally, Cluster 4 shows two dominant temporal characters: high future-orientation, and high past-positive orientation. Present-hedonism is also high, but the cluster still cannot be characterized with a balanced TP, because present-fatalism has the highest mean score of all clusters, and the past-negativity achieves the second highest mean across the clusters.

**Table 4:** Descriptive results of the hierarchical Cluster analysis.

Variables	Clusters				
	1	2	3	4	5
*N*	86	55	70	56	79
Age, mean(*SD*)	59.14(15.83)	33.42(15.51)	33.57(18.54)	54.04(20.49)	34.10(14.66)
Gender, women/men	60/26	45/10	47/23	38/18	56/23
Education, mean(*SD*)	3.91(1.25)	4.60(0.99)	4.59(0.97)	3.46(1.46)	4.91(0.48)
*Time Perspectives*, mean(*SD*)					
– Past negative	1.91(.87)	3.32(0.69)	3.47(0.83)	3.37(1.03)	1.82(0.48)
– Past positive	3.69(1.01)	2.82(0.76)	3.5(0.80)	4.50(0.47)	2.72(0.80)
– Present fatalism	3.33(1.04)	2.33(0.77)	3.05(0.94)	3.41(1.15)	2.06(0.72)
– Present hedonism	1.79(0.62)	1.89(0.60)	3.26(0.96)	3.32(0.90)	2.83(0.81)
– Future	4.64(0.43)	3.65(0.06)	3.03(0.78)	4.62(0.39)	3.75(0.78)
Machiavellianism, mean(*SD*)	3.05(0.58)	3.15(0.48)	3.18(0.63)	3.09(0.63)	2.89(0.64)
Narcissism, mean(*SD*)	2.22(0.64)	2.74(0.80)	2.94(0.75)	2.70(0.75)	3.04(0.63)
Psychopathy, mean(*SD*)	1.73(0.54)	2.04(0.58)	2.37(0.67)	2.03(0.61)	2.07(0.57)

A one-way ANOVA revealed a significant effect of clustering on each of the DT traits (Machiavellianism: *F*(4,345) = 3.92, *p* < 0.01; narcissism: *F*(4,345) = 15.56, *p* < 0.001, psychopathy: *F*(4,345) = 11.87, *p* < 0.001). Of the demographic variables, while age (*F*(4,345) = 40.73, *p* < 0.001) and education (*F*(4,345) = 20.34, *p* < 0.001) were found to be significantly different across the clusters, a *χ*^2^ test revealed no significant difference in gender among the five clusters (*χ*^2^ = 3.97, *n.s.*).

Corrected multiple comparisons revealed that older participants belonged mainly to Cluster 1 and Cluster 4. Participants’ age in these clusters was significantly different from that in Clusters 2, 3, and 5 (C1 vs. C2: *t*(139) = 9.48, *p* < 0.001; C1 vs. C3: *t*(154) = 9.29, *p* < 0.001; C1 vs. C5: *t*(63) = 10.51, *p* < 0.001; C4 vs. C2: *t*(109) = 5.97, *p* < 0.001; C4 vs. C3: *t*(124) = 5.88, *p* < 0.001; C4 vs. C5: *t*(133) = 6.6, *p* < 0.001). Both of these clusters have an imbalanced TP profile. In Cluster 1, where the mean age was found to be the highest, participants have a high future orientation along with high past-positivity, but with low present-hedonistic, and high present-fatalistic attitudes. Cluster 4 is similar to Cluster 1, but here past-negativity and present-hedonism also have a high level. Participants’ mean education level was the highest in Cluster 5, which was significantly different from Cluster 1 (*t*(163) = 6.68, *p* < 0.001) and Cluster 4 (*t*(133) = 7.12, *p* < 0.001), and marginally different from Cluster 3 (*t*(147) = 2.54, *p* = 0.052). Cluster 5 has the most balanced TP profile, suggesting that more educated individuals reported a more balanced profile of TP.

The lowest cluster mean for Machiavellianism was found in the most balanced cluster, Cluster 5, suggesting an imbalanced TP profile of individuals having high scores on this DT trait. After correction for multiple comparisons, Machiavellianism scores in Cluster 5 were significantly lower than those in Cluster 2 (*t*(132) = -2.51, *p* < 0.05), Cluster 3 (*t*(147) = -2.73, *p* < 0.05), and Cluster 4 (*t*(133) = -3.30, *p* < 0.01). None of the other clusters was significantly different for Machiavellianism.

The imbalanced TP profile of individuals with high scores on psychopathy received support from the finding that the highest mean score for psychopathy was obtained in Cluster 3. This score was significantly higher than that of each of the other clusters including, importantly, the most balanced cluster, Cluster 5 (Cluster 1: *t*(154) = 6.55, *p* < 0.001; Cluster 2: *t*(123) = 2.84, *p* < 0.05; Cluster 4: *t*(124) = 3.60, *p* < 0.001; Cluster 5: *t*(147) = 2.85, *p* < 0.05). This Cluster 3 cannot be characterized with a dominant time perspective (please see the details above), but, for example, future orientation has the lowest mean in this cluster, which is in line with the expectation that, compared to other individuals, individuals high on psychopathy are concerned more about the past and present and less about the future.

Finally, in contrast with the other two DT traits, the highest cluster mean of narcissism was found in Cluster 5, with the most balanced TP profile. After adjustment, this score, however, was significantly different only from the scores obtained in Cluster 1 (*t*(163) = 7.7, *p* < 0.001). Individuals belonging to Cluster 1 were older than individuals in the other clusters, and were characterized with low present-hedonism, high present-fatalism, high past-positive orientation as well as with high concern about future.

## Discussion

In the present study, we investigated whether the temporal attitudes of individuals scoring high on the DT traits were significantly different from an optimal, balanced profile (BTP). Importantly, the age of participants covered a long and continuous period of adulthood—from young adulthood to old age—in order to examine the moderation effect of age on DT – BTP associations. We used two operationalization methods to quantify BTP: the deviation from BTP model defining optimal points on each TPs (DBTP; Stolarski et al., 2011), and a person-oriented approach relying on a hierarchical cluster analysis (Boniwell et al., 2010).

The results of both the DBTP and person-oriented analyses clearly suggested that Machiavellianism and psychopathy were found to be similar in that they both showed considerable deviation from the time perspective profile considered to be balanced. The analyses of the absolute deviation from the optimal level on each TP indicated that these two traits shared a similar non-optimal profile in terms of viewing the present as being controlled by external events (i.e., present fatalism). In addition, individuals with higher scores on psychopathy tended to deviate from the optimum in terms of negative attitudes toward the past (i.e., past-negativity) and consideration of the future (i.e., future orientation). The results of the cluster analysis refines the conclusion about the association of psychopathy with future TP further by showing that more psychopathic individuals’ concern about the future is significantly less (see Cluster 3) than that of individuals having a more BTP profile (i.e., Cluster 5). Previous studies have suggested that underlying the temporal attitude of individuals reporting higher Machiavellian and psychopathic character is their negative early life experiences, resulting a fast life strategy later in adulthood (e.g., Birkás and Csathó, 2015; Moraga et al., 2017). The present results about the non-optimal temporal attitude of Machiavellianism and psychopathy extends these earlier findings by implying that the generally biased temporal attitude of these traits might reach a functionally unbeneficial level. As we discussed in the Section “Introduction,” a non-optimal time perspective profile holds an increased risk of many adverse health and social consequences. In line with this, Jonason et al. (2015) found that Machiavellianism and psychopathy were linked to undesirable psychological and physical health conditions. In addition, psychopathy was associated with a diminished subjective life expectancy. This latter finding is consistent with the low and non-optimal level of future-related attitudes found in both the present and previous studies for individuals with higher psychopathic personality. Jonason et al. (2015) interpreted the negative health outcomes as the associated costs of fast life history strategies characterizing DT traits. The current findings might raise the possibility that the deviation from a BTP profile is an important mediator between DT traits and impaired health outcomes. Future studies might consider the examination of this mediating role of DBTP in this context.

Present hedonism represented the only exception of time perspective scales with a closer-to-optimal value for individuals who were high on psychopathy. In the context of previous studies this finding is not surprising, because several studies have reported a positive association between hedonistic attitudes and psychopathy (e.g., Birkás and Csathó, 2015; Kajonius et al., 2015; Moraga et al., 2017). For example, Kajonius et al. (2015) examined the universal social values of the DT traits, and found that individuals higher on psychopathy were more likely to rate hedonistic values (e.g., pleasure and enjoying life) highly. Here, however, we showed that although hedonism might be a universal characteristic of individuals high on psychopathy, their level of hedonism might still be optimal or even more optimal than the average level of the population. This finding suggests further that a more pronounced psychopathic character prevents individuals from dysfunctionally low present and reward oriented thoughts. Also, conversely, if the level of present hedonism is around the optimal, then individuals might still not be threatened by the adverse consequences of excessive hedonism, for example, by an increased risk of substance use (Keough et al., 1999).

In contrast to Machiavellianism and psychopathy, narcissism showed a negative association with DBTP. The more BTP for more narcissistic individuals is in line with the notion that narcissism is different from other DT traits in terms of more facets of their behavioral profile (e.g., Jonason et al., 2015). To take one example, unlike those high in Machiavellianism or psychopathy, individuals high in narcissism tend to show higher engagement in social activity. Compared with the other DT traits, they have been found to report more reasons to form friendships, such as, for example, shared interests and intelligence (Jonason and Schmitt, 2012). Life satisfaction and well-being seem also to be high for individuals with higher narcissistic personality (Rose and Campbell, 2004). In line with these points, the more narcissistic participants in Jonason et al.’s (2015) study indicated better physical and mental health outcomes than those who had a lower narcissism score, suggesting a buffering role of narcissism against impaired health conditions.

The time perspective profile of individuals high in narcissism, with a more positive shade and with a more flexible character, might provide a strong basis for this buffer function. Importantly, however, our DBTP analysis suggested an age dependency in the association between narcissism and BTP. That is, narcissism predicted a more BTP only in elderly individuals. This suggests that a buffering function of narcissism against impaired health and/or everyday dysfunctionality might become more important with increasing age. The results reported here (see, e.g., the cluster analysis) imply that through aging people come to view life through a different lens than in the younger ages, one of higher past-positivism, higher present-fatalism, and lower present hedonism. These changes seem to be manifested in an imbalanced time perspective profile. A part of these changes, especially those related to enhanced present fatalism and reduced present hedonism might be compensated for by special personality characteristics such as narcissism. To consider this argument further, narcissism might act to reduce the effect of an imbalanced TP profile on aging-related health impairments. Future studies might consider addressing this question specifically.

Age-related effects on the associations between BTP and DT were not found to be moderated by gender. In relation to the participants’ gender, only two effects were identified. First, we found the same as many other studies did, that is, men reported higher scores on Machiavellianism and psychopathy than women (see e.g., Jonason et al., 2013; Jonason and Davis, 2018). This gender or sex difference is usually explained with the generally faster life history strategy of men as compared to women (e.g., Furnham et al., 2013). Second, men showed a lower deviation from BTP than women did, a finding which was underlined mainly by a lower deviation from the optimal present-hedonism level for men. This finding suggests again that an elevated level of present-hedonism is not necessarily significantly above an optimal level.

In addition to age and gender, the effect of participants’ level of education on BTP was investigated. Generally, the deviation from a balanced TP profile was found to be independent of participants’ education level. Only a higher deviation from the optimal level on past-positivity was obtained for individuals having a higher education degree. This finding is rather surprising, and it might require further examination. In the present study, we considered the factor of education simply as a degree reached by individuals across the education levels. In contrast, future studies might consider education in a wider socioeconomic context.

The present study has some limitations. First, the internal consistency of the subscales of SD3 was only just acceptable. Second, no time window was defined for the consideration of time-periods. Thus, we do not know whether participants’ answers referred to distant or near time. This could limit both the interpretation of age-related changes in TP and our understanding of the moderating effects of age, especially in comparisons between young and elderly individuals. Third, we did not differentiate between primary and secondary psychopathy or between grandiose and vulnerable narcissism. These dimensions might, however, have different associations with the time-perspectives. Fourth and last, the dataset was biased in relation to the education level of participants: higher education was much more common among the younger than the older participants. Although we controlled for education in the analyses, the education bias might still unduly influence our conclusions to some degree. Future studies might therefore also consider examining how number of years spent in education affects associations between the DT traits and time orientation attitudes.

In spite of the above limitations, the present study supplies evidence for the general conclusion that among the three DT traits, Machiavellianism and psychopathy were associated with an increased deviation from the time perspective profile suggested to be optimal. In contrast, higher scores on narcissism were associated with a more balanced view about time. The DBTP analysis, however, suggested that this beneficial effect of narcissism is prevalent only among elderly individuals.

## Ethics Statement

This study was carried out in accordance with the recommendations of APA and the Regional Ethics Committee of the University of Pécs with written informed consent from all subjects. All subjects gave written informed consent in accordance with the Declaration of Helsinki. The protocol was approved by the ‘Regional Ethics Committee of the University of Pécs.’

## Author Contributions

BB and AC designed and performed the study, analyzed the data, and wrote the paper. AM interpreted the data and wrote the paper. All authors discussed the results and implications and commented on the manuscript at all stages.

## Conflict of Interest Statement

The authors declare that the research was conducted in the absence of any commercial or financial relationships that could be construed as a potential conflict of interest.

## Footnotes

1 http://www.thetimeparadox.com/surveys/
